# Group I mGluR antagonist rescues the deficit of D1-induced LTP in a mouse model of fragile X syndrome

**DOI:** 10.1186/1750-1326-7-24

**Published:** 2012-05-28

**Authors:** Zhao-Hui Xu, Qi Yang, Bin Feng, Shui-bing Liu, Nan Zhang, Jiang-hao Xing, Xiao-Qiang Li, Yu-mei Wu, Guo-Dong Gao, Ming-Gao Zhao

**Affiliations:** 1 Department of Neurosurgery, Tangdu Hospital, Fourth Military Medical University, 17 Changle West Road, Xi’an, 710032, China; 2 Department of Pharmacology, School of Pharmacy, Fourth Military Medical University, 17 Changle West Road, Xi’an, 710032, China

**Keywords:** Group I mGluRs, Dopamine, Long-term potentiation prefrontal cortex

## Abstract

**Background:**

Fragile X syndrome (FXS) is caused by the absence of the mRNA-binding protein Fragile X mental retardation protein (FMRP), encoded by the *Fmr1* gene. Overactive signaling by group 1 metabotropic glutamate receptor (Grp1 mGluR) could contribute to slowed synaptic development and other symptoms of FXS. Our previous study has identified that facilitation of synaptic long-term potentiation (LTP) by D1 receptor is impaired in *Fmr1* knockout (KO) mice. However, the contribution of Grp1 mGluR to the facilitation of synaptic plasticity by D1 receptor stimulation in the prefrontal cortex has been less extensively studied.

**Results:**

Here we demonstrated that DL-AP3, a Grp1 mGluR antagonist, rescued LTP facilitation by D1 receptor agonist SKF81297 in *Fmr1*KO mice. Grp1 mGluR inhibition restored the GluR1-subtype AMPA receptors surface insertion by D1 activation in the cultured *Fmr1*KO neurons. Simultaneous treatment of Grp1 mGluR antagonist with D1 agonist recovered the D1 receptor signaling by reversing the subcellular redistribution of G protein-coupled receptor kinase 2 (GRK2) in the *Fmr1*KO neurons. Treatment of SKF81297 alone failed to increase the phosphorylation of NR2B-containing N-methyl D-aspartate receptors (NMDARs) at Tyr-1472 (p-NR2B-Tyr1472) in the cultures from KO mice. However, simultaneous treatment of DL-AP3 could rescue the level of p-NR2B-Tyr1472 by SKF81297 in the cultures from KO mice. Furthermore, behavioral tests indicated that simultaneous treatment of Grp1 mGluR antagonist with D1 agonist inhibited hyperactivity and improved the learning ability in the *Fmr1*KO mice.

**Conclusion:**

The findings demonstrate that mGluR1 inhibition is a useful strategy to recover D1 receptor signaling in the *Fmr1*KO mice, and combination of Grp1 mGluR antagonist and D1 agonist is a potential drug therapy for the FXS.

## Background

Fragile X syndrome is the most common form of inherited mental retardation, characterized by moderate to severe mental retardation, attention deficits, and anxiety. This disease results from the expansion of a trinucleotide repeat (CGG) within the X-linked FMR1 gene [1-4]. As a result of this expansion, the product of the FMR1 gene, fragile X mental retardation protein (FMRP), is not expressed [1,5,6]. FMRP is a mRNA binding protein that is associated with polyribosomes and is thought to be involved in the translational efficiency and/or trafficking of certain mRNAs [7,8].

Recent progress in the study of the mouse model of Fragile X has led to a theory that the absence of FMRP leads to misregulation of protein synthesis at the synapse that occurs in response to mGluR activity [9,10]. An extension of this theory is that modulation of mGluR activity with antagonists should reverse phenotypes attributable to loss of FMRP function. Exaggerated translation linked to group 1 (Grp 1) mGluRs, i.e., overactive signaling by Grp 1 mGluRs, could contribute to slowed synaptic development and other symptoms of FXS [9,10]. This is supported by the observations that mGluR-induced long-term depression is increased in the hippocampus of *Fmr1* knock-out (KO) mice [9].

A myriad of studies have defined synaptic plasticity deficits in *Fmr1* KO mice [11-15]. Long-term potentiation (LTP), a type of long lasting synaptic plasticity, is believed to be involved in learning and memory [16,17]. Prefrontal cortex (PFC)-associated molecular, cellular, and behavioral abnormality in *Fmr1* KO mouse is a useful model for testing the efficacy of therapeutic strategies aimed at treating the cognitive impairments in FXS [18]. Our previous studies show that long-term potentiation (LTP) is completely abolished in the PFC [11]. Dopamine (DA) in the prefrontal cortex (PFC) plays a critical role in cognitive functions and neuropsychiatric pathology [19-23]. It is well known that DA functions in its target cells through five subtypes of DA receptors (D1-5) [22,24,25]. Recent studies have been carried out by numerous groups to investigate the cellular mechanism for DA modulation in PFC neurons [21,22,26-30]. Our recent study reveals that FMRP contributes to dopamine modulation of AMPA GluR1 receptor synaptic insertion and dopaminergic facilitation of LTP [31]. These findings provide the evidence that FMRP acts as a key messenger for DA receptor-mediated modulation in forebrain neurons.

Given the enhanced mGluR activity in the brains of *Fmr1* KO mice, we explored the possibility that mGluR1 misregulation might act on dopamine modulation in the prefrontal synaptic plasticity. Here we showed that mGluR1 inhibition rescued LTP facilitation by D1 receptor in *Fmr1* KO mice, without having effects on basal glutamatergic synaptic transmission.

## Results

### Grp1 mGluR antagonist rescues LTP facilitation by D1 activation in F*mr1* KO mice

**Figure 1:**
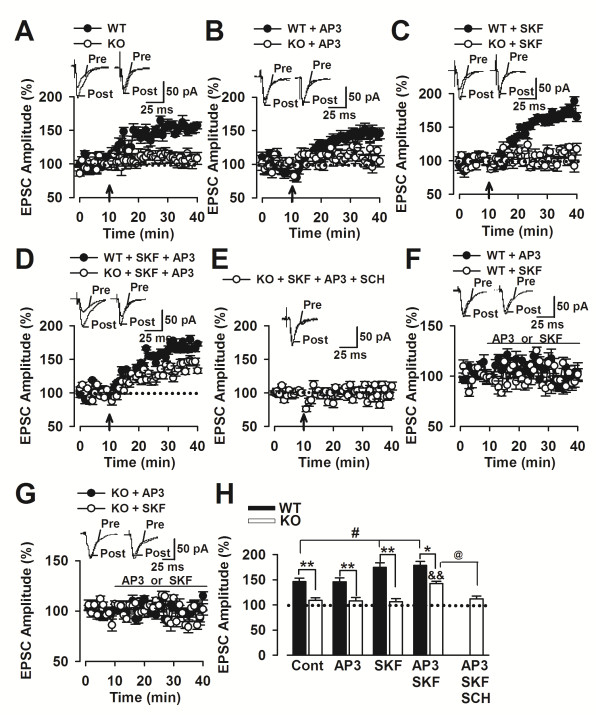
**Rescue of D1-induced LTP by DL-AP3 in the*Fmr1*KO mice. (A)** The pairing training produced a significant, long-lasting potentiation of synaptic responses in *Fmr1* WT mice (n = 12 slices/5 mice), but not in *Fmr1* KO mice (n = 9 slices/4 mice); **(B)** DL-AP3 (10 μM) did not alter the amplitude of LTP in *Fmr1* WT mice (n = 9 slices/3 mice). DL-AP3 (10 μM) failed to induce LTP in *Fmr1* KO mice (n = 11 slices/5 mice). **(C)** SKF81297 (5μM) facilitated LTP induction in *Fmr1* WT mice (n = 8 slices/3 mice), but failed to induce LTP in *Fmr1* KO mice (n = 11 slices/5 mice). **(D)** Bath application of SKF81297 (5 μM) and DL-AP3 (10 μM) induced LTP in *Fmr1* WT mice (n = 10 slices/3 mice) (n = 10 slices/3 mice) and markedly rescued the LTP induction by SKF81297 in the *Fmr1* KO mice (n = 12 slices/4 mice). **(E)** SCH23390 (10 μM) blocked the LTP by synergistic application of SKF81297 and DL-AP3 in the KO mice (n = 13 slices/4 mice). **(F and G)** SKF81297 (5 μM, 30 min) or DL-AP3 (10 μM, 30 min) had no effect on basal synaptic responses without pairing training (n = 8) in the *Fmr1* WT and KO mice. **(H)** Summary of the effects of DL-AP3 or/and SKF81297 on the LTP induction. * *p* < 0.05, ** *p* < 0.01 compared with WT; # *p* < 0.05 compared with control; ^&&^*p* < 0.01 compared with SKF81297 + DL-AP3 in WT mice; ^@^*p* < 0.05 compared with SKF81297 + DL-AP3 in KO mice.

The PFC, including its cingulate region, plays an important role in learning and memory, drug addiction, and pain [11,32,33]. First, we performed whole-cell patch-clamp recordings in visually identified pyramidal neurons in layers II–III of cingulate region of PFC slices. LTP was induced by pairing presynaptic stimulation with postsynaptic depolarization. The pairing training produced a significant, long-lasting potentiation of synaptic responses in WT mice (146.5% ± 6.7%, n = 12 slices/5 mice; *p* < 0.01 versus baseline; Figure 1A), but not in KO mice (108.9% ± 5.4%, n = 9 slices/4 mice; *p* > 0.05 versus pairing training only; Figure 1A). Since Grp1 mGluR-LTD is exaggerated in the *Fmr1* KO mouse, we next examined the effects of mGluR1 antagonist on LTP induction in the PFC. It has been reported that high dose of mGluR1 antagonist, DL-2-amino-3-phosphonopropionic acid (DL-AP3, 300 μM) or (+)-alpha-methyl-4-carboxyphenylglycine (MCPG, 500 μM), reduced homosynaptic LTP in the hippocampus [34,35]. In the present study, the slices were incubated with mGluR1 antagonist DL-AP3 at low dose of 10 μM at least for 30 min before the LTP induction was performed. At the concentration of 10 μM, DL-AP3 did not alter the amplitude of LTP as compared to the pairing training only in the WT (145.7% ± 7.9%, n = 9 slices/3 mice; *p* < 0.05 versus pairing training only; Figure 1B) and KO (108.3% ± 6.2%, n = 11 slices/5 mice; *p* < 0.05 versus pairing training only; Figure 1B) mice. We next paired D1 agonist application with an LTP induction protocol. Bath application of SKF81297 (5 μM) for 10 min paired with the LTP induction protocol significantly enhanced the amplitude of LTP (174.3% ± 9.4%, n = 8 slices/3 mice; *p* < 0.01 versus pairing training only; Figure 1C), suggesting that D1 receptor activation can facilitate LTP induction in the WT mice. However, in *Fmr1* KO mice SKF81297 pairing training could not induce LTP (106.2% ± 6.2%, n = 11 slices/5 mice; *p* > 0.05 versus baseline responses; Figure 1C), demonstrating that dopaminergic facilitation of LTP is impaired in *Fmr1* KO mice.

Next, D1 agonist and mGluR1 antagonist were applied simultaneously to detect their synergistic effects on the LTP induction. Bath application of SKF81297 (5 μM) and DL-AP3 (10 μM) for 10 min induced a significant LTP (178.5% ± 8.1%, n = 10 slices/3 mice; *p* < 0.01 versus pairing training only; Figure 1D). Further comparison analysis found that the amplitude of the LTP was similar to the LTP induced by SKF81297 (5 μM) alone in the WT mice (*p* > 0.05 as compared to the SKF81297 + DL-AP3). However, simultaneous application of DL-AP3 (10 μM) markedly rescued the LTP induction by SKF81297 in the *Fmr1* KO mice (137.6% ± 6.5%, n = 12 slices/4 mice; *p* < 0.05 versus SKF81297 alone; Figure 1D and 1H). Additional D1 receptor antagonist SCH23390 (10 μM) for 10 min prior to, and during electrical stimulation totally blocked the LTP which was rescued by synergistic application of SKF81297 and DL-AP3 in the KO mice (102.8% ± 6.5%, n = 13 slices/4 mice; *p* > 0.05 versus baseline; Figure 1E and 1H).

SKF81297 (5 mM, 30 min) or DL-AP3 (10 μM, 30 min) had no effect on basal synaptic responses (Figure 1F and 1G). Thesis data indicate that lower dose of DL-AP3 (10 μM) dose not alter the LTP induction in the *Fmr1* WT and KO mice; however, it rescues the LTP facilitation of D1 receptor activation in the *Fmr1* KO mice.

### Adenylyl cyclase agonist rescues LTP facilitation by D1 activation in F*mr1* KO mice

**Figure 2:**
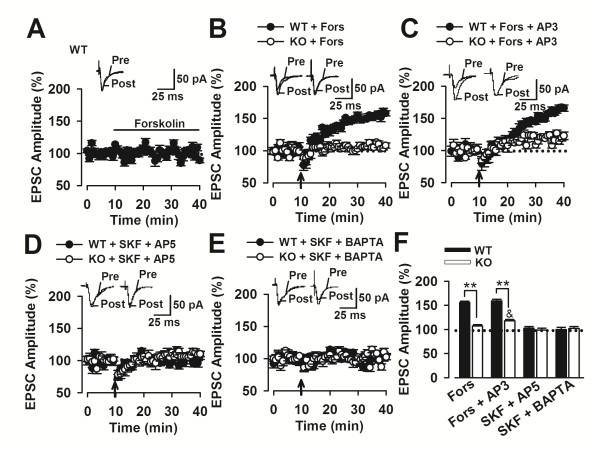
**Synergistic effects of DL-AP3 and forskolin on the LTP induction in the*Fmr1*KO mice. (A)** Forskolin (10 μM) did not induce chemical LTP in the ACC without pairing training (n = 9 slices/4 mice). **(B)** Forskolin (10 μM) failed to induce LTP in the *Fmr1* KO mice (n = 8 slices/4 mice). LTP in the presence of forskolin (10 μM) is similar to the control in the *Fmr1* WT mice (n = 12 slices/4 mice). **(C)** Bath application of SKF81297 (5μM) and DL-AP3 (10 μM) induced similar LTP as control in *Fmr1* WT mice (n = 10 slices/4 mice). Combining forskolin with DL-AP3 partially rescued LTP in the *Fmr1* KO mice (n = 11 slices/4 mice). **(D)** LTP facilitation by SKF81297 (5 μM) was blocked by AP5 (50 μM) (n = 10 slices/3 mice). **(E)** LTP facilitation by SKF81297 (5 μM) was blocked by 10 mM BAPTA in the pipette solution (n = 10 slices/3 mice). **(F)** Summary of the effects of forskolin or/and DL-AP3 on the LTP induction. * *p* < 0.05, ** *p* < 0.01 compared with WT; ^&^*p* < 0.05 compared with forskolin in KO mice.

In our previous study, we found that D1 receptor signaling is impaired, i.e., the increase in cAMP caused by SKF81297 is attenuated, accompanied by D1 receptor hyperphosphorylation at serine sites in the PFC of *Fmr1* KO mice. To detect whether or not the increase of cAMP could mimic the function of D1 receptor, the adenylyl cyclase activator, forskolin, was used. Many studies have used forskolin to stimulate AMPAR trafficking and induce chemical LTP [36-39]. In the present study, unlike in the hippocampus, LTP was not induced during the application of forskolin (10 μM), which was not paired with the electrical stimulation in the ACC, i.e., no chemical LTP was induced in the ACC (101.9% ± 3.6%, n = 9 slices/4 mice; *p* > 0.05 versus baseline before perfusion of forskolin; Figure 2A). The amplitude of LTP is similar to that of the control when the forskolin was paired with the electrical stimulation in the *Fmr1* WT mice (155.8% ± 3.5%, n = 12 slices/4 mice; *p* > 0.05 versus pairing training only; Figure 2B). Combining forskolin with DL-AP3 did not result in an increase in the amplitude of LTP compared with the case, in which the forskolin alone was used in the *Fmr1* WT mice (159.4% ± 3.4%, n = 10 slices/4 mice; *p* > 0.05 versus forskolin alone; Figure 2C). Forskolin (10 μM) paired with the electrical stimulation was unable to induce LTP in the *Fmr1* WT mice (107.2% ± 3.2%, n = 8 slices/4 mice; *p* > 0.05 versus pairing training only; Figure 2B). However, combining forskolin with DL-AP3 partially rescued LTP in the *Fmr1* KO mice, even if the amplitude was only 118% ± 2.8% of the base line (n = 11 slices/4 mice; *p* < 0.05 versus DL-AP3 alone; Figures 2C and 2F).

To determine whether or not dopaminergic modulation of LTP required NMDAR activation, we applied a selective NMDAR antagonist, AP5 (50 μM), and found that LTP facilitation by SKF81297 (5 μM) was completely blocked (Figure 2D). Similarly, LTP was abolished by 10 mM BAPTA in the pipette solution (Figure 2E), indicating that dopaminergic modulation of LTP depended on the activation of NMDARs and elevated postsynaptic Ca^2+^ concentrations.

### Grp1 mGluRs modulate plasticity by a postsynaptic mechanism

**Figure 3:**
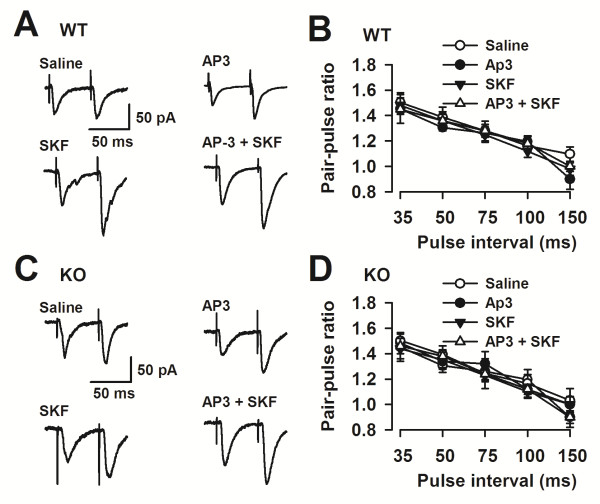
**Postsynaptic mechanisms underlying the LTP facilitation by Grp1 mGluRs inhibition.** Paired-pulse facilitation (the ratio of EPSC2/EPSC1) was recorded with intervals of 35, 50, 75, 100, and 150 ms. **(A)** Representative traces of PPF with an interval of 50 ms recorded in the *Fmr1* WT mice ACC. **(B)** PPF was not changed at each interval in *Fmr1* WT mice after perfusion of DL-AP3 (10 μM) or/and SKF81297 (5 μM) (n = 6 slices/3 mice in each group). **(C)** Representative traces of PPF with an interval of 50 ms recorded in the *Fmr1* KO mice ACC. **(D)** PPF was not changed at each interval in *Fmr1* KO mice after perfusion of DL-AP3 (10 μM) or/and SKF81297 (5 μM) (n = 6 slices/3 mice in each group).

In the different regions of the hippocampus, both presynaptic and postsynaptic mechanisms have been proposed to contribute to the expression of LTP [40]. To determine whether presynaptic and/or postsynaptic mechanisms are involved in Grp1 mGluRs modulation of LTP, we measured paired-pulse facilitation (PPF) in the ACC. PPF is a phenomenon by which a second synaptic stimulation of equal magnitude evokes a larger synaptic response than the first, and has been used as a tool to implicate presynaptic probability of transmitter release [41,42]. As shown in Figure 3, PPF induced at five different intervals did not differ in WT (Figure 3A) and *Fmr1* KO mice (Figure 3B) in the presence of DL-AP3 (10 μM) or/and SKF81297 (5 μM) in the ACC. The data suggest that presynaptic mechanisms do not seem to be involved in the Grp1 mGluRs modulation of LTP.

### No changing of basal glutamatergic synaptic transmission by Grp1 mGluRs inhibition

**Figure 4:**
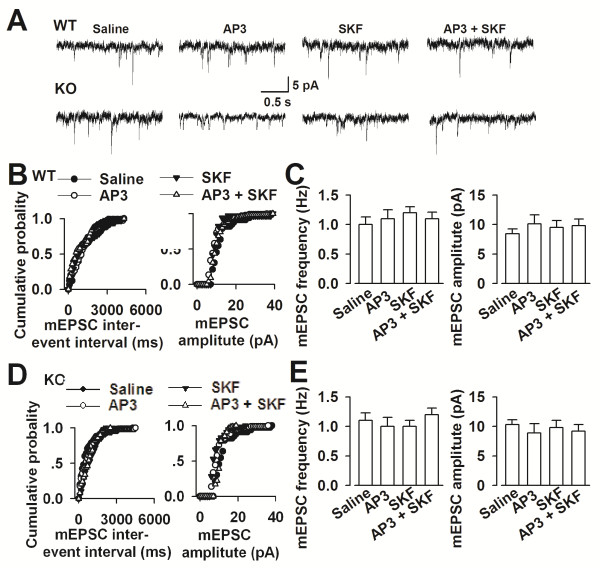
**Basal glutamatergic synaptic transmission in the ACC. (A)** AMPA receptor-mediated mEPSCs recorded in the ACC neurons at a holding potential of −70 mV. Representative traces show the mEPSCs in slices treated with saline, DL-AP3 or/and SKF81297. **(B)** Cumulative frequency (left) and amplitude (right) histogram of the mEPSCs from the *Fmr1* WT mice. **(C)** Summary of mEPSCs frequency (left) and amplitude (right) in neurons from the saline, DL-AP3 (10 μM, n = 7 slices/3 mice), SKF81297 (5 μM, n = 6 slices/3 mice), and DL-AP3 (10 μM) + SKF81297 (5 μM) (n = 7 slices/3 mice). **(D)** Cumulative frequency (left) and amplitude (right) histogram of the mEPSCs from the *Fmr1* KO mice. **(E)** Summary of mEPSCs frequency (left) and amplitude (right) in neurons from the saline, DL-AP3 (10 μM, n = 6 slices/3 mice), SKF81297 (5 μM, n = 6 slices/3 mice), and DL-AP3 (10 μM) + SKF81297 (5 μM) (n = 7 slices/3 mice).

To determine whether Grp1 mGluRs inhibition affects basal glutamatergic synaptic transmission in the ACC, we measured the miniature AMPA receptor-mediated EPSCs (mEPSCs). As shown in Figure 4, no significant alteration was detected in the AMPA receptor-mediated mEPSCs frequency and amplitude among the control, DL-AP3 (10 μM) or/and SKF81297 (5 μM) treated slices from *Fmr1* WT and KO mice (n = 8 in each group, Figure 4). Results indicate that by Grp1 mGluRs inhibition and activation of D1 receptor have no effect on the basal excitatory synaptic transmission in the ACC.

### Inhibition of Grp1 mGluRs rescues expression of GluR1 by D1 receptor

**Figure 5:**
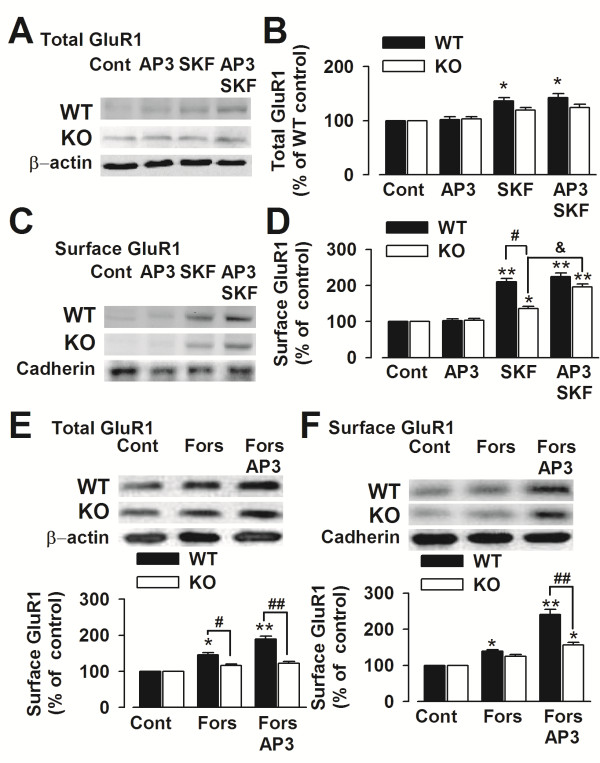
**Expression of GluR1 in cultured cortical neurons. (A)** Representatives of western blot to detect the total expression of AMPA receptor GluR1 subunit. **(B)** Total expression of GluR1 was increased after treatment with SKF81297 (5 μM) alone or combining with DL-AP3 (10 μM) in the neurons form *Fmr1* WT mice (n = 4 dishes). **(C)** Representatives of western blot to detect the surface expression of AMPA receptor GluR1 subunit. **(D)** Surface expression of GluR1 was increased after treatment with SKF81297 (5 μM) alone or combining with DL-AP3 (10 μM) in neurons from *Fmr1* WT and KO mice. **(E)** Forskolin (10 μM) alone or combining with DL-AP3 (10 μM) increased the total expression levels of GluR1 in the cultures from *Fmr1* WT mice but not from KO mice. **(F)** Surface expression of GluR1 was increased after treatment with forskolin (10 μM) alone or combining with DL-AP3 (10 μM) in neurons from *Fmr1* WT and KO mice. n = 4 dishes. * *p* < 0.05, ** *p* < 0.01 compared with control; # *p* < 0.05, ## *p* < 0.01 compared between *Fmr1* WT and KO mice; ^&^*p* < 0.05 compared between the neurons treated with SKF81297 and DL-AP3 + SKF81297 in *Fmr1* KO mice.

To determine the synergistic effect on the AMPA receptor expression and surface insertion, we treated cultured PFC neurons with the D1 receptor agonists SKF81297 and Grp1 mGluRs antagonist DL-AP3. SKF81297 (5 μM) alone or combining with DL-AP3 (10 μM) increased the total expression levels of GluR1 in the cultures from *Fmr1* WT mice (Figure 5A). However, neither SKF81297 nor DL-AP3 affected the total expression levels of GluR1 in cultures from *Fmr1* KO mice (Figure 5B). To further investigate the signaling involved in the synaptic plasticity, we tested the effects of DL-AP3 or/and SKF81297 on AMPA receptor GluR1 subunits surface trafficking in cultured PFC neurons. Surface expression of GluR1 was increased after treatment with SKF81297 (5 μM) alone or combining with DL-AP3 (10 μM) in the cultures from *Fmr1* WT mice (Figure 5C). Contrast to the unchanged total levels of GluR1, surface expression of GluR1 was increased after simultaneous treatment of SKF81297 (5 μM) and DL-AP3 (10 μM) in the cultures from *Fmr1* KO mice (Figure 5D). This finding demonstrates that inhibition of Grp1 mGluRs can rescue the surface expression of AMPA GluR1 receptors by D1 receptor in *Fmr1* KO PFC neurons.

Furthermore, the synergistic effects of DL-AP3 and forskolin on AMPA receptor GluR1 subunits surface trafficking were detected in cultured PFC neurons. Forskolin (10 μM) alone or combined with DL-AP3 (10 μM) increased the total expression levels of GluR1 in the cultures from *Fmr1* WT mice (Figure 5E). However, neither forskolin (10 μM) nor DL-AP3 affected the total expression levels of GluR1 in cultures from *Fmr1* KO mice (Figure 5E). The surface expressions of GluR1 in the cultures from *Fmr1* WT mice increased after treatment with forskolin (10 μM) alone and combined with DL-AP3 (10 μM) (Figure 5F). In contrast with the unchanged total levels of GluR1, surface expressions of GluR1 in the cultures from *Fmr1* KO mice increased after simultaneous treatment of forskolin (10 μM) and DL-AP3 (10 μM) (Figure 5F). This finding demonstrated the synergistic effects on the surface expressions of AMPA GluR1 receptors in *Fmr1* KO PFC neurons resulting from the inhibition of Grp1 mGluRs and activation of adenylyl cyclase.

### Inhibition of Grp1 mGluRs reverses redistribution of GRK2

**Figure 6:**
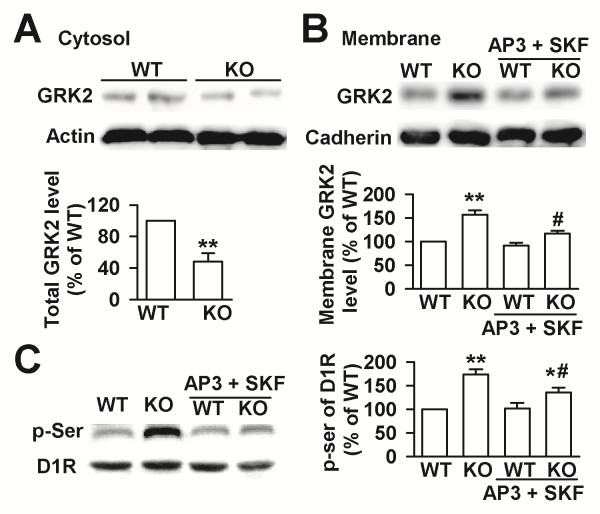
**Expression of GRK2 and D1 receptor phosphorylation.** Expression levels of GRK2 were decreased in cytosol preparation **(A)** and increased in membrane preparation **(B)** from the cultures of *Fmr1* KO mice as compared with KO neurons. Synergistic addition of DL-AP3 (10 μM) and SKF81297 (5 μM) reversed redistribution of GRK2 between membrane and cytosol in *Fmr1* KO neurons. **(C)** Synergistic addition of DL-AP3 (10 μM) and SKF81297 (5 μM) reduced the increase of D1 receptor phosphorylation at serine sites to a level similar to WT neurons. n = 4 dishes. * *p* < 0.05, ** *p* < 0.01 compared between *Fmr1* WT and KO neurons; # *p* < 0.05 compared with KO control.

Our previous study shows that D1 receptor signaling is impaired, accompanied by D1 receptor hyperphosphorylation at serine sites and subcellular redistribution of G protein-coupled receptor kinase 2 (GRK2) in the prefrontal cortex of the *Fmr1* KO mice. FMRP interactes with GRK2, and pharmacological inhibition of GRK2 rescues D1 receptor signaling in Fmr1 KO neurons [31]. D1 receptors can be phosphorylated by GRKs [36]. We found that GRK2 levels were increased in the cell membrane but decreased in the cytosol in the culture neurons from *Fmr1* KO mice (Figure 6A). Synergistic addition of DL-AP3 (10 μM) and SKF81297 (5 μM) reversed redistribution of GRK2 between membrane and cytosol in *Fmr1* KO neurons (Figure 6B). Consistently, synergistic addition of DL-AP3 (10 μM) and SKF81297 (5 μM) reduced the increase of D1 receptor phosphorylation at serine sites to a level similar to WT neurons (Figure 6C). Treatment of SKF81297 (5 μM) alone did not reduce the level of D1 receptor phosphorylation (data not shown). These data implied that inhibition of Grp1 mGluRs reversed redistribution of GRK2 and decreased the D1 receptor phosphorylation at serine sites.

### D1 receptor, mGluR5, and NMDARs expression in the PFC cultures

**Figure 7:**
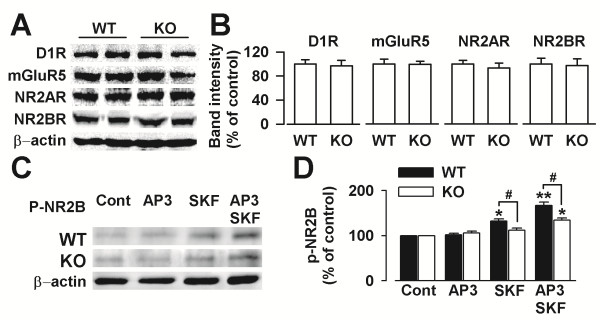
**Expression of D1 receptor, mGluR5, and glutamate receptor subunits. (A)** Representatives of western blot from the prefrontal cortex of *Fmr1* WT and KO mice. **(B)** There were no differences in expressions of D1 receptor, mGluR5, NR2A, and NR2B between the WT and *Fmr1* KO mice ACC. n = 4 mice per group. **(C)** Representatives of western blot to detect the level of phosphorylation of NR2B subunit at Tyr-1472 (p-NR2B-Tyr1472). **(D)** Level of p-NR2B-Tyr1472 was increased after treatment with SKF81297 (5 μM) alone or combining with DL-AP3 (10 μM) in neurons from *Fmr1* WT and KO mice. n = 4 dishes. * *p* < 0.05, ** *p* < 0.01 compared with control; # *p* < 0.05 compared between *Fmr1* WT and KO mice.

To further explore the synaptic mechanisms behind the impairment of dopaminergic modulation of LTP, we examined the expression of mGluR5 and D1 receptor in the ACC from WT and *Fmr1*KO mice. Even though the level of D1 receptor phosphorylation was increased, the total D1 expression was not changed in the *Fmr1* KO mice (Figure 7A). There were no difference in the levels of mGluR5, NMDA receptor NR2A subunit, and NR2B subunit between the WT and *Fmr1* KO mice (Figure 7A and 7B). Thus, the impairment of mGluR5 and D1 receptor modulation of synaptic potentiation is not due to defect in levels of mGluR5, D1 receptor, and NR2A- or NR2B- containing NMDA receptor.

The function of NMDARs is regulated by its phosphorylation. Given that tyrosine phosphorylation of NMDARs contributes to LTP [43,44], we examined the tyrosine phosphorylation levels of NR2B subunits with anti-phosphotyrosine antibody to determine whether or not NMDARs are phosphorylated to SKF81297 and DL-AP3. We found that the presence of SKF81297 (5 μM) alone or SKF81297 (5 μM) plus DL-AP3 (10 μM) did not affect total NR2A or NR2B subunit expression in the cultures from *Fmr1* WT and KO mice (Figure 7B). However, SKF81297 (5 μM) alone or SKF81297 (5 μM) plus DL-AP3 (10 μM) significantly increased the level of phosphorylation of the NR2B subunit at Tyr-1472 (p-NR2B-Tyr1472) in the cultures from *Fmr1* WT mice (Figure 7C). Meanwhile, SKF81297 (5 μM) alone failed to increase p-NR2B-Tyr1472 in the cultures from *Fmr1* KO mice. Synergistic addition of DL-AP3 (10 μM) rescued the p-NR2B-Tyr1472 by SKF81297 (5 μM) in the cultures from *Fmr1* KO mice (Figure 7D). These data implied that the increase of p-NR2B-Tyr1472 contributed to the surface expression of GluR1 by SKF81297 and DL-AP3 in the KO cultures.

### Synergistic effect of simultaneous treatments on the behavior of *Fmr1* KO mice

**Figure 8:**
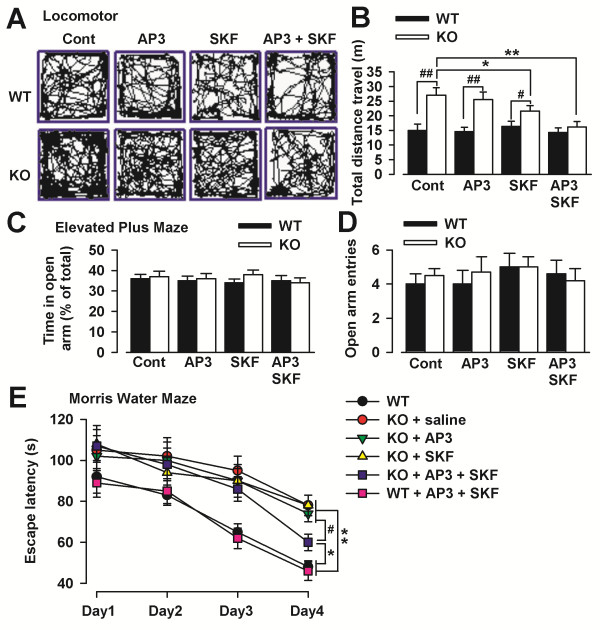
**Effect of simultaneous treatments on behavior in*Fmr1*KO mice. (A)** Sample traces of locomotor activity in the open-field test for control, SKF81297, or/and DL-AP3 injected WT or *Fmr1* KO mice, respectively. **(B)***Fmr1* KO mice traveled more than WT mice at basal condition (n = 7 mice for WT, n = 7 mice for Fmr1 KO). SKF81297 (1 mg/kg body weight, injected subcutaneously) significantly reduced the distance traveled by *Fmr1* KO mice (n = 6 mice for control, n = 8 mice for SKF81297). DL-AP3 (4 mg/kg) did not affect locomotor activity in *Fmr1* KO mice; however, DL-AP3 combining SKF81297 significantly reduced the distance traveled in a larger scale as compared with the SKF81297 injection alone (n = 7 mice). SKF81297 or/and DL-AP3 did not affect locomotor activity in WT mice. **(C)** Time in the open arms had no differences between *Fmr1* KO and WT mice in the mice treated with SKF81297 (1 mg/kg) alone or plus DL-AP3 (4 mg/kg). **(D)** Open arm entries had no differences between *Fmr1* KO and WT mice in the mice treated with SKF81297 (1 mg/kg) alone or plus DL-AP3 (4 mg/kg). N = 6 mice in each group. **(E)** Simultaneous treatment of SKF81297 (1 mg/kg) alone or plus DL-AP3 (4 mg/kg) reduced the swim latency on the final day in the *Fmr1* KO mice. N = 6 mice in each group. * *p* < 0.05, ** *p* < 0.01 compared with *Fmr1* WT mice. # *p* < 0.05 compared between the mice treated with SKF81297 and DL-AP3 + SKF81297 in *Fmr1* KO mice.

Fragile X patients and animal models exhibit behavioral phenotypes that are consistent with deficits in PFC function, such as hyperactivity, anxiety, and learning abnormalities (Bear et al., 2004; Moon et al., 2006; Ventura et al., 2004). We analyzed locomotor activity, anxiety, and learning abilities to characterize hyperactivity and learning deficit, the common phenotypes in fragile X syndrome. We found increased locomotor activity in *Fmr1* KO mice (Figure 8A), which was consistent with the our previous study [31]. The locomotor activity was not affected following SKF81297 (1 mg/kg) or/and DL-AP3 (4 mg/kg) subcutaneous injection after forty-five minutes in WT mice (Figure 8B). However, application of the D1 receptor agonist SKF81297 or simultaneous treatment of SKF81297 and DL-AP3 significantly reduced the distance traveled by *Fmr1* KO mice (Figure 8B). These data indicate that *Fmr1* KO mice exhibit hyperactivity and that simultaneous treatment of D1 agonist and Grp1 mGluRs antagonist can reduce the hyperactivity phenotype.

We then investigated anxiety-like behaviors with the elevated plus-maze test. We found no differences between *Fmr1* KO and WT mice and no effect of SKF81297 (1 mg/kg) alone or plus DL-AP3 (4 mg/kg) (Figure 8C and Figure 8D). These data indicate that anxiety-like behaviors are not detectable in this fragile X mouse model and suggest that these behaviors are probably not related to the D1 receptor signaling pathway. These findings are consistent with our previous study [31].

Last, the learning ability was detected in the Morris Water Maze test. Mice were treated with SKF81297 (1 mg/kg i.p.) alone or plus DL-AP3 (4 mg/kg i.p.) for 5 weeks. Swim latency was compared over the 4 days of the experiment, and there were significant differences in escape latency between genotype at day 1. SKF81297 or DL-AP3 treatment alone did not change the swim latency in the *Fmr1* WT and KO mice. Simultaneous treatment of SKF81297 or DL-AP3 also did not improve the learning ability in the *Fmr1* WT mice. However, Simultaneous treatment of SKF81297 and DL-AP3 reduced the swim latency on the final day in the *Fmr1* KO mice (Figure 8E). Moreover, we compared the swimming speed during the water maze test and found no differences among the groups (data not shown). These findings clearly show that simultaneous treatment of SKF81297 and DL-AP3 significantly improves the spatial leaning of *Fmr1* KO mice.

## Discussion

In present study, we have clearly demonstrated that prefrontal LTP in slices from *Fmr1* knock-out mice is severely attenuated compared with LTP in slices from *Fmr1* WT mice. Although a previous study has shown a strong reduction in neocortical LTP, the nature of this decrease was not tested [11]. Here, we first showed that inhibition of Grp1 mGluRs rescued LTP facilitation by D1 receptor in *Fmr1* KO mice, without effects on basal synaptic transmission. Then, using the culture systems we found that Grp1 mGluR inhibition enhanced the GluR1-subtype AMPA receptors surface insertion by D1 activation in the cortical neurons from *Fmr1* KO mice. Last, behavioral tests indicated that simultaneous treatment of D1 agonist and Grp1 mGluRs antagonist could reduce the hyperactivity phenotype of *Fmr1*KO mice.

### Exaggerated signaling by grp 1 mGluRs in the FXS

Recent progress in the study of the mouse model of Fragile X has led to a theory that the absence of FMRP leads to misregulation of protein synthesis at the synapse that occurs in response to mGluR activity [9,10]. An increase in mGluR5-mediated activity leads to an increased LTD of synaptic activity in the *Fmr1*KO mice. Exaggerated translation linked to Grp 1 mGluRs, i.e. overactive signaling by Grp 1 mGluRs, could contribute to slowed synaptic development and other symptoms of FXS [9,10]. Our previous study has shown a strong reduction of LTP in the prefrontal cortex [11], a structure involving in higher brain functions such as learning and memory, drug addiction, and attention (Courtney et al., 1998; Frankland et al., 2004; Zhao et al., 2005b; Zhuo, 2002, 2008). In the present study, we found that low dose of mGluR1 antagonist DL-AP3 did not affect the LTP induction in the prefrontal cortex and the AMPA surface trafficking in the culture neurons. However, this weak inhibition of Grp 1 mGluRs could rescue the synaptic plasticity mediated by dopamine receptor. This finding suggests a complex relationship between mGluRs and dopamine receptors. The therapeutic window for mGluR1 antagonists may be narrow because mGluR1 is essential for normal cerebellar function.

### Dopaminergic modulation of long-term synaptic plasticity

In the prefrontal cortex, including the ACC, activation of the NR2B and NR2A subunits of the NMDA receptor is critical for the induction of cingulate long-term synaptic plasticity [45-47]. Dopamine D1 receptor regulates NMDA receptor functions by direct protein-protein interactions [48,49]. D1 receptor activation has been reported to increase the surface expression of AMPA receptors and facilitate their synaptic insertion in the cultured neurons [26,50,51]. The surface expression of AMPA receptors correlates with their synaptic insertion [50-52]. In the present study, we found that D1 receptors facilitated cingulate prefrontal LTP through increasing synaptic incorporation of homomer GluR1 in the *Fmr1* WT mice. However, the plasticity and the increase of GluR1 surface expression induced by stimulating D1 receptor was significantly attenuated in *Fmr1* KO neurons. This indicates that silence of *Fmr1* gene plays as a key role in the deficits of DA-mediated modulation of excitatory transmission in forebrain neurons [31].

### Adenylyl cyclase activation and synaptic plasticity in the FXS

It is reported that patients with FXS show abnormal function or regulation of the catalytic subunit of adenylate cyclase. They found that in patients with FXS basal production of cAMP was 63% of that of control subjects [53]. Our previous study reports that D1 receptor signaling was impaired, i.e., the increase in cAMP caused by SKF81297 was attenuated, accompanied by D1 receptor hyperphosphorylation at serine sites in the PFC of Fmr1 KO mice [31]. In the present study, we found that simultaneous application of adenylyl cyclase activator forskolin with DL-AP3 could partially rescued LTP in the *Fmr1* KO mice (Figure 2C and 2F) in the slices recording. Consistent with this result, surface expression of GluR1 was increased after simultaneous treatment of forskolin (10 μM) and DL-AP3 (10 μM) in the cultures from *Fmr1* KO mice (Figure 5F). These findings suggest that activation of adenylyl cyclase by forskolin or D1 receptor is a potential therapy strategy for the FXS.

### Grp 1 mGluRs, D1 modulation of AMPA receptors, and synaptic Potentiation

Here, we found that the decreased LTP in the prefrontal cortex observed in *Fmr1* KO mice correlates with decreased surface levels of GluR1 expression. Our present recordings from the adult brain slice revealed that D1 receptors facilitated cingulate prefrontal LTP through increasing synaptic incorporation of homomer GluR1, even though low dose of mGluR1 antagonist DL-AP3 did not affect the LTP induction in the prefrontal cortex and the AMPA surface trafficking in the culture neurons. Unaltered PPF suggests that inhibition of Grp 1 mGluRs rescue the LTP induction in *Fmr1* KO mice by D1 receptor in a postsynaptic manner. However, we cannot rule out the possible presynaptic modulation of inhibitory transmission by dopamine receptor and Grp 1 mGluRs [54,55], since inhibitory transmission was blocked in our recordings. We failed to find consistent D1 modulation of basal AMPA receptor transmission in adult slice recordings [56], while in cultured PFC neurons, D1 receptor activation caused GluR1 subunit surface expression and synaptic insertion. The inconsistency between adult slices and cultured neurons could be explained by developmental differences (cultured neurons are liable to express proteins that may not be expressed *in vivo*) or the differences between *in vivo* and *in vitro* neuronal networks [57-60].

### GRK2 and D1 receptor hyperphosphorylation in the FXS

G-protein coupled receptors can be desensitized following activation by agonists by becoming phosphorylated by members of the family of G protein-coupled receptor kinases (GRKs). Phosphorylated receptors are then bound by arrestins, which prevent further stimulation of G proteins and downstream signaling pathways [36]. Our present and previous studies show that D1 receptor signaling is impaired, accompanied by D1 receptor hyperphosphorylation at serine sites and subcellular redistribution of G protein-coupled receptor kinase 2 (GRK2) in the prefrontal cortex of the *Fmr1* KO mice. Synergistic application of DL-AP3 and SKF81297 could reverse redistribution of GRK2 between membrane and cytosol in *Fmr1* KO neurons and further reduce the increase of D1 receptor phosphorylation at serine sites to a level similar to WT neurons. These results suggest that D1 receptor hyperphosphorylation might be caused by the subcellular redistribution of GRK2 in the *Fmr1* KO mice and that inhibition of Grp1 mGluRs might modulate the subcellular distribution of GRK2. These data implied that inhibition of Grp1 mGluRs reversed redistribution of GRK2 and decreased the D1 receptor phosphorylation at serine sites. Thus, inhibition of Grp1 mGluRs can recover the capability of D1 receptor to activate G proteins and downstream signaling pathways.

### NMDA receptor and synaptic transmission in *Fmr1* KO mice

In addition, there is no difference in D1 receptor, mGluR5, NR2A, and NR2B expression between *Fmr1* KO and WT mice as shown by western blot. The results suggest that the impairment of mGluR5 and D1 receptor facilitation of LTP in prefrontal cortex of *Fmr1* KO mice is not due to defect in levels of mGluR5, D1 receptor, and NR2A- or NR2B- containing NMDA receptor. The function of NMDARs is regulated by its phosphorylation and tyrosine phosphorylation of NMDARs is thought to contribute to LTP [43,44]. In this study, we found that activation of D1 significantly increased the level of phosphorylation of NR2B subunit at Tyr-1472 (p-NR2B-Tyr1472) in the cultures from *Fmr1* WT mice but not in the cultures from *Fmr1* KO mice. Synergistic inhibition of Grp1 mGluR could rescue the p-NR2B-Tyr1472 by D1 receptor in the cultures from *Fmr1* KO mice. Thus, the increase of phosphorylation of NMDARs by Grp1 mGluR antagonist may contribute to the improvement of surface expression of GluR1 and synaptic plasticity by D1 activation in the *Fmr1* KO mice.

In summary, the present study, together with our previous study, reveals that mGluR1 inhibition is a useful strategy to recover D1 receptor signaling in the *Fmr1*KO mice, and that combination of Grp1 mGluR antagonist and D1 agonist is a potential drug therapy for the FXS.

## Materials and methods

### Animal

Male mice aged 5 to 6 weeks were used for behavioral experiments and brain slice recordings. *Fmr1* KO mice (strain name: FVB.129P2- *Fmr1*^tm1Cgr^/J; stock #:4624) and control wild-type (WT) mice on the same strain background (stock # 4828) were purchased from The Jackson Laboratory (Genetics Research, Bar Harbor, Maine, USA) and bred in the animal facility of the Fourth Military Medical University. All mice were housed in plastic boxes in groups of 4 under a 12:12 light cycle; food and water were provided *ad libitum*. The use of animals was reviewed and approved by the Institutional Ethical Committee of the Fourth Military Medical University.

### Slice preparation

Coronal prefrontal brain slices (300 μM) from *Fmr1* WT and KO mice, containing the anterior cingulate cortex (ACC), were prepared using standard methods [45]. Slices were transferred to submerged recovery chamber with oxygenated (95% O_2_ and 5% CO_2_) artificial cerebrospinal fluid (ACSF) containing (in mM: 124 NaCl, 2.5 KCl, 2 CaCl_2_, 1 MgSO_4_, 25 NaHCO_3_, 1 NaH_2_PO_4_, 10 glucose) at room temperature for at least 1 h.

### Whole-cell recordings

Experiments were performed in a recording chamber on the stage of an Axioskop 2FS microscope with infrared DIC optics for the visualization of whole-cell patch clamp recording. In the ACC layer II to III, pyramidal neurons have been reported as the major locations of intra-cortical horizontal pathways [61]. The superficial layers of the ACC receive substantial glutamate input [62]. In the present study, we recorded excitatory postsynaptic currents (EPSCs) from neurons in layer II to III induced by the stimulation of layer V. AMPA receptor (AMPAR)-mediated EPSCs were induced by repetitive stimulations at 0.02 Hz, and neurons were voltage clamped at −70 mV. We identified pyramidal neurons by injecting depolarized currents into neurons to induce action potentials. The typical firing pattern of a pyramidal neuron showed significant firing frequency adaptation, whereas the pattern in an interneuron showed fast-spiking action potentials followed by pronounced hyperpolarization. After obtaining stable EPSCs for at least 10 min, LTP was induced by 80 pulses at 2 Hz, and then paired with postsynaptic depolarization at + 30 mV (i.e., “pairing training”). In calculating the LTP amplitude, the last 5 min recordings (6 EPSCs) before the pairing training were averaged as the baseline. The last 5 min recordings (6 EPSCs) before the ending were also averaged in order to analyze the changes of LTP. The recording pipettes (3 to 5 MΩ) were filled with solution containing (mM) 145 K-gluconate, 5 NaCl, 1 MgCl_2_, 0.2 EGTA, 10 HEPES, 2 Mg-ATP, and 0.1 Na_3_-GTP (adjusted to pH 7.2 with KOH). Picrotoxin (100 μM) was always present to block GABA_A_ receptor-mediated inhibitory synaptic currents. To record the mEPSCs, 0.5 μM Tetrodotoxin (TTX) was added into the bath solution. The access resistance was 15 to 30 MΩ, which was monitored throughout the experiment. Data were discarded if access resistance changed by over 15% during an experiment.

### Primary culture of prefrontal cortical neurons

Primary cultures of prefrontal cortical neurons were prepared from embryonic 18 days old (E18) Sprague–Dawley rats as described previously [31]. Serum-free B27/neurobasal medium (Invitrogen, Carlsbad, USA CA) was used to prevent glial cell growth which is reduced to less than 0.5% of the nearly pure neuronal population, as judged by immunocytochemistry for glial fibrillary acidic protein and neuron-specific enolase [63]. Cells were seeded at the density of 3 ~ 4 × 10^3^ cells/cm^2^ onto 24-well plates coated with 50 μg/ml poly-D-lysine (Sigma, St. Louis, MO). The cultures were incubated at 37°C in 95% air, 5% CO_2_ with 95% humidity. Half of the medium was replaced every 4 days. Cultures were used for experiments between 10 and 14 days *in vitro* (DIV). SKF81297 (5 μM), DL-AP3 (10 μM), and forskolin (5 μM) were added in the culture medium for 24 h before the cells were harvested.

### Western blot analysis

Western blot analysis was performed as described previously [31]. Equal amounts of protein (50 μg) from the cultures were separated and electrotransferred onto PDVF membranes (Invitrogen), which were probed with antibodies for mGluR5 (1:500, Abcam, Cambridge, MA), GluR1 (1:300, Abcam), NR2A (1:200, Millipore), NR2B (1:1000, Millipore), p-NR2B Tyr1472 (1:1000, Cell signaling), GRK2 (1:1000, Santa Cruz), D1 receptor (1:1000, Millipore), FMRP (1:1000, Millipore), anti-phosphoserine of D1 receptor (1:2500, BD Biosciences), with β-actin (1:10000, Sigma) or cadherin (1:2000, Sigma) as loading control. For data quantitation, band intensity was expressed relative to the loading control (β-actin or cadherin). The membranes were incubated with horseradish peroxidase conjugated secondary antibodies (anti-rabbit/anti-mouse IgG for the primary antibodies), and bands were visualized using an ECL system (Perkin Elmer).

### Surface biotinylation assay

Surface protein samples were detected through a biotinylation assay, followed by Western blot analysis with the antibodies as previously described [31]. Cell monolayers were washed and incubated in sulfo-NHS-LC-biotin (0.3 mg/ml in cold PBS; Pierce, Rockford, IL) for 30 min. Surface biotinylation was stopped by removing the solution, followed by incubation in 10 mM ice-cold glycine in PBS for 20 min. Cells were washed thrice with cold PBS and lysed by RIPA buffer containing (mM) 20 HEPES pH 7.4, 100 NaCl, 1 EGTA, 1 Na3VO4, 50 NaF, 1 mM 4-(2-aminoethyl)-benzenesulfonylfluoride hydrochloride, plus 1% NP-40,1% deoxycholate, 0.1%SDS, 10 mg/ml leupeptin, and 1 mg/ml aprotinin. Biotinylated proteins were precipitated with 100 ml of ImmunoPure Immobilized Streptavidin (Pierce, Rockford, IL), separated on 4% to 12% SDS-PAGE gels, and transferred to polyvinylidene fluoride membranes. The membranes were probed with anti-GluR1. The membranes were incubated with horseradish peroxidase-conjugated secondary antibodies (anti-rabbit/anti-mouse IgG for the primary antibodies), and bands were visualized using an ECL system (Perkin Elmer).

### Locomotor activity

Horizontal locomotor activity was conducted, as described in the report [64], with an open-field (OP) test system (Jiangliang, Shanghai, China). The open field was a square arena (30 cm × 30 cm × 30 cm) with clear Plexiglas walls and floor, and then placed inside an isolation chamber with dim illumination and a fan. Mice were placed in the center of the box, allowed to freely explore for 10 min, and videotaped using a camera fixed above the floor and then analyzed with a video-tracking system. Forty-five minutes after the subcutaneous injection of saline, DL-AP3, and/or SKF81297, each subject was placed in the center of the open field, and its activity was measured for 30 min.

### Elevated plus maze

The Elevated Plus Maze (EPM) was conducted as described in the report [64]. The apparatus comprised of two open arms (25 × 8 × 0.5 cm) and two closed arms (25 × 8 × 12 cm) that extended from a common central platform (8 × 8 cm). The apparatus elevated to a height of 50 cm above the floor. Mice were allowed to habituate to the testing room for 2 days before the test, and pretreated with gentle handling two times per day to eliminate their nervousness. For each test, individual animals were placed in the center square, facing an open arm, and allowed to move freely for 5 min. Mice were videotaped using a camera fixed above the maze and analyzed with a video-tracking system. Open and closed arm entries (all four paws in an arm) were scored by an experienced observer. The number of entries and time spent in each arm were recorded.

### Morris water maze test

In the Morris Water Maze test, mice were treated either with SKF81297 (1 mg/kg i.p.) alone or with SKF81297 plus DL-AP3 (4 mg/kg i.p.) for 5 weeks. The experiments were conducted as described previously [65]. The maze was made of white opaque plastic with a diameter of 120 cm and 40 cm high walls, which was filled with water at 25°C to avoid hypothermia. A small escape platform (diameter = 10 cm) was placed at a fixed position in the center of one quadrant 25 cm from the perimeter and was hidden 1 cm beneath the water surface. The room contained a number of fixed visual cues on the walls. The acquisition trial phase consisted of 4 training days (days 1 to 4), with 2 trials per day and a 15-min inter-trial intervals. Four points equally spaced along the circumference of the pool (north, south, east, and west) served as the starting positions, which were randomized across the four trials each day. If an animal did not reach the platform within 90 s, it was guided to the platform, where it had to remain for 30 s before being returned to its home cage. Mice were kept dry between trials in a plastic holding cage filled with paper towels. Starting locations were changed every trial. The results of four trials were averaged and used in the data analysis. The time required to escape onto the hidden platform was recorded as escape latency.

### Data analysis

Results were expressed as mean ± SEM. Statistical comparisons were performed by one-way analysis of variance (ANOVA) using the post-hoc comparisons with Tukey's test. In all cases, *p* < 0.05 was considered statistically significant.

## Abbreviations

DA, Dopamine; EPM, Elevated Plus Maze; EPSC, Excitatory postsynaptic current; FMRP, Fragile X mental retardation protein; FXS, Fragile X syndrome; Grp1 mGluR, group 1 metabotropic glutamate receptor; KO, Knockout; LTP, Long-term potentiation; NMDA, N-methyl D-aspartate; PFC, Prefrontal cortex; WT, Wild type.

## Competing interests

The author(s) declare that they have no competing interests.

## Authors' contributions

ZHX, QY, and BF carried out cell culture and electrophysiology. SBL, NZ, JHX, XQL, and YMW carried out western-blot and behavioral test. GDG and MGZ were responsible for experimental design and writing the manuscript. All authors read and approved the final manuscript.
